# Supplementary material for: Impact of the COVID-19 pandemic on intra-household gender disparities in the Middle East and North Africa region: A scoping review protocol
Source: PLoS One. 2024 Nov 18;19(11):e0313838. doi: 10.1371/journal.pone.0313838 (PMC11573154; doi:10.1371/journal.pone.0313838)
Supplement: S1 Appendix — (DOCX) [file pone.0313838.s002.docx]

**Supporting Information**

**Appendix 1. Search Strategy**

**MEDLINE (Ovid)**

| 1 | coronaviridae/ or coronavirus/ or exp Coronavirus Infections/ or exp Betacoronavirus/ |
| --- | --- |
| 2 | (pneumonia/ or pneumonia, viral/ or exp Viruses/) and (exp Disease Outbreaks/ or exp Epidemiology/ or Epidemiology.fs.) |
| 3 | (coronavirus/ or betacoronavirus/ or coronavirus infections/) and (disease outbreaks/ or epidemics/ or pandemics/) |
| 4 | (betacoronavir* or beta-coronavir* or coronavir* or COVID* or "corona vir*").ti,ab. |
| 5 | 1 or 2 or 3 or 4 |
| 6 | limit 5 to yr="2019 -Current" |
| 7 | exp COVID-19/ or exp COVID-19 Testing/ or COVID-19 Vaccines/ or SARS-CoV-2/ |
| 8 | (COVID19 or COVID-19 or nCov* or "CoV 2" or CoV2 or 2019ncov* or 2019-ncov* or 19nCoV* or 19-ncov* or 2019cov* or 2019-cov* or HCoV* or "novel CoV").ti,ab. |
| 9 | ((novel or new or "19" or "2019" or wuhan or huanan or hubei or china or chinese) adj3 (coronavir* or "corona vir*" or betacoronavir* or "beta corona*" or CoV or COVID* or "severe acute respiratory" or SARS* or pneumonia)).ti,ab. |
| 10 | ((coronavir* or "corona vir*" or betacoronavir* or "beta corona*" or cov*) adj3 (pandemic* or epidemic* or outbreak* or crisis or crises or disease*)).ti,ab. |
| 11 | (SARS2 or SARS-CoV-2 or SARSCOV-2 or SARS-COV2 or SARSCOV2 or (SARS adj2 (coronavir* or "corona vir*")) or "Severe Acute Respiratory Syndrome corona*").ti,ab. |
| 12 | (longCOVID* or "long COVID*" or postCOVID* or "post COVID*" or postcoronavir* or "postcorona vir*" or "post corona vir*" or "post coronavir*" or postSARS* or "post sars*" or "wuhan virus" or "wuhan pneumonia").ti,ab. |
| 13 | 7 or 8 or 9 or 10 or 11 or 12 |
| 14 | 6 or 13 |
| 15 | domestic violence/ or spouse abuse/ or gender-based violence/ or exp intimate partner violence/ or Emotional Abuse/ or Physical Abuse/ or Family Conflict/ |
| 16 | Disease/ or exp Mental Disorders/ or Depression/ or Mood Disorders/ or Fear/ or Anxiety/ or Psychological Distress/ |
| 17 | exp Health/ |
| 18 | personal autonomy/ or professional autonomy/ or Adaptation, Psychological/ or dependency, psychological/ or Codependency, Psychological/ |
| 19 | exp Social Discrimination/ or Racism/ or Sexism/ or Sexual Harassment/ |
| 20 | Child Care/ or Primary Health Care/ |
| 21 | decision making/ or decision making, shared/ |
| 22 | Household Work/ or exp Family Characteristics/ |
| 23 | Gender Equity/ or Feminism/ or empowerment/ or Women's Rights/ |
| 24 | Unemployment/ or exp Income/ or exp Health Expenditures/ |
| 25 | exp morbidity/ or exp mortality/ |

| 26 | exp Food Security/ or exp Food Insecurity/ |
| --- | --- |
| 27 | (abuse* or abusi* or anxiet* or anxious* or autonom* or carework* or "care work*" or "child car*" or childcar* or child-car* or coping or adjust* or (psycholog* adj2 adapt*) or decision-making or "decision making" or dependen* or independen* or codependen* or "co dependen*" or co-dependen* or interdependen* or discriminat* or distress* or stress* or "domestic work*" or empower* or fear or feminis* or (food adj2 insecurit*) or (food adj2 securit*) or gender* or harass* or ((mental* or reproduc* or sex* or physical*) adj2 (health* or illness* or disorder* or disease*)) or (expenditure* adj2 health*) or (expense* adj2 health*) or "home school*" or homeschool* or home-school* or household* or "house hold*" or housework or housekeeping or chore or chores or income* or wage or wages or salary or salaries or payment* or pay or pays or injustice or ((interpartner* or partner* or spous* or marital or domestic or family) adj2 (violen* or conflict*)) or (job adj2 loss*) or mistreat* or maltreat* or ill-treat* or illtreat* or "ill treat*" or morbidit* or mortalit* or oppress* or racism or racist* or sexis* or (preventive adj2 care) or (primary adj2 care) or unemploy* or "unpaid care" or "un-paid care" or "nonpaid care" or "non-paid care" or ((wom?n* or female*) adj2 (status* or position* or right*))).ti,ab. |
| 28 | 15 or 16 or 17 or 18 or 19 or 20 or 21 or 22 or 23 or 24 or 25 or 26 or 27 |
| 29 | algeria/ or egypt/ or libya/ or morocco/ or tunisia/ or djibouti/ or somalia/ or sudan/ or iraq/ or jordan/ or lebanon/ or syria/ or yemen/ |
| 30 | (iraq* or jordan* or lebanon or lebanese or yemen* or algeria* or egypt* or libya* or morocc* or tunis* or sudan* or palestin* or gaza or "west bank" or "east* jerusalem" or djibouti* or djibuti* or somal* or syria*).ti,ab. |
| 31 | 29 or 30 |
| 32 | 14 and 28 and 31 |
| 33 | limit 32 to yr="2020 -Current" |
