# Supplementary material for: Impact of the COVID-19 pandemic on intra-household gender disparities in the Middle East and North Africa region: A scoping review protocol
Source: PLoS One. 2024 Nov 18;19(11):e0313838. doi: 10.1371/journal.pone.0313838 (PMC11573154; doi:10.1371/journal.pone.0313838)
Supplement: S2 Appendix — (DOCX) [file pone.0313838.s003.docx]

**Supporting Information**

**Appendix 2. Data Extraction Instrument**

| Study Information | | | | Study Design and Methodology | | | | | | | |
| --- | --- | --- | --- | --- | --- | --- | --- | --- | --- | --- | --- |
| Study title | Author | Year | Journal | Qualitative vs Quantitative vs Mixed | Study design | Country | Study setting | Sample size | Sampling method | Data collection method | Data analysis method |

| Participant Characteristics | | | | | | Intra-Household Gender Inequality Measures | | | |
| --- | --- | --- | --- | --- | --- | --- | --- | --- | --- |
| Gender | Age | Marital Status | Education Level | Socioeconomic Status | Other relevant information (refugees, etc.) | Changes in Gender Roles | Division of Household Tasks | Economic Dependence | Access to Resources and Services |

| Intra-Household Gender Inequality Measures | | | Contextual Factors | | | Quality Assessment | Conclusions and Implications | |
| --- | --- | --- | --- | --- | --- | --- | --- | --- |
| Power Dynamics | Gender-Based Violence | Health outcomes (SRH, physical, etc.) | Cultural Norms | Government Policies | Public Health Measures | Limitations | Key conclusions of the study | Implications for policy or practice |
