# Supplementary material for: Impact of the COVID-19 pandemic on intra-household gender disparities in the Middle East and North Africa region: A scoping review protocol
Source: PLoS One. 2024 Nov 18;19(11):e0313838. doi: 10.1371/journal.pone.0313838 (PMC11573154; doi:10.1371/journal.pone.0313838)
Supplement: Attachment — Submitted filename: R2 - Protocol_Response to Reviewers.docx [file pone.0313838.s004.docx]

#### Journal: PLOS One

PONE-D-24-14476

*15-10-2024*

Dear Editor,

On behalf of all co-authors, we would like to thank you and the reviewers for the time spent on reviewing our work, as well as the valuable comments that helped us improve the quality of our manuscript titled “***Impact of the COVID-19 Pandemic on Intra-household Gender Disparities in the Middle East and North Africa region: A Scoping Review Protocol***”. All comments have been addressed, as can be seen in the point-by-point response to the comments by reviewers below.

All authors confirm that the manuscript is not under consideration by any other journal and that authors have no conflict of interest. All authors have read and approved the submission of this revised manuscript.

Yours sincerely,

Jocelyn DeJong, PhD

Associate Provost and Professor at the Faculty of Health Sciences, American University of Beirut, Beirut, Lebanon

**Email**: [Jd16@aub.edu.lb](mailto:Jd16@aub.edu.lb)

**Journal requirements**

*1. Please review your reference list to ensure that it is complete and correct. If you have cited papers that have been retracted, please include the rationale for doing so in the manuscript text, or remove these references and replace them with relevant current references. Any changes to the reference list should be mentioned in the rebuttal letter that accompanies your revised manuscript. If you need to cite a retracted article, indicate the article’s retracted status in the References list and also include a citation and full reference for the retraction notice.*

Thank you for the comment. The references have been revised.

**Reviewer #1**

We would like to thank the Reviewer for the time reviewing our work and the valuable comments.

*Reviewer #1: This is a review of a revised scoping study protocol. I think the authors have given careful consideration to the comments from myself and another reviewer, which have significantly improved the intended scoping review. I think this has the potential to be a very important study, providing a much needed contribution to the literature. I would be happy for the authors to proceed, with just two minor comments.*

*As noted by R2, the first mention of LMIC should be spelled out fully. This currently takes place on p4, but is not spelled out until p5.*

Thank you for your comment. The manuscript has been edited accordingly (p. 4, line 106).

*On p7, the authors provide a careful outline of the themes to be included in the review. However, no references are provided. These should be included to understand the basis for including these themes.*

Thank you for your valuable feedback. We have incorporated relevant references on page 7 to support the themes outlined in the review. These citations provide a clear rationale for the selection of these themes, drawing on key studies and existing literature that highlight their importance (Lines 176-255).

**Reviewer #2**

We would like to thank the Reviewer for the time spent on our work and the valuable comments.

*Reviewer #2: The revised versions is largely responsive to my original comments. I'll discuss each in turn, numbered in the same way.*

*1. Dealt with appropriately.*

Thank you for your comment.

*2. This is a positive change and should increase the yield of useable studies.*

Thank you for your comment.

*3. These are much improved. I was only seeking clarification on what each theme was intended to cover, and the new text also provides a rationale for doing so, but I see the other reviewer requested this so I am satisfied with it.*

Thank you for your comment.

*4. I appreciate that there may be a paucity of longitudinal studies and that it is therefore reasonable to include cross-sectional studies with proper acknowledgement of their limits in capturing change. Having said that, I am still a little concerned about how change will be inferred in this review, as the inclusion criteria make no reference to studies having to be about change (just "impact", which is vaguer). Besides using the word "impact" the only relevant inclusion statement is "contribute directly to understanding gender disparities within households during the pandemic", which doesn't entail any reference to change during or as a result of the pandemic, real or perceived. Studies that merely contribute to understanding disparities in the pandemic's present (disparities that may have been pre-existing) will therefore meet inclusion criteria. So I remain a little uncertain how the findings will illuminate change unless the included studies at the very least examine perceived change from before to during (or after) the pandemic. That doesn't currently seem to be a strict requirement of included studies, so I remain a little doubtful how the review will clarify change or impact unless the inclusion criteria are tightened a little.*

Thank you for your insightful feedback regarding our inclusion criteria and the inference of change in our review. In light of your comments, we have refined our research question and themes to ensure a clearer focus on both the characterization of the present data and the identification of studies that report change related to intra-household gender disparities during the COVID-19 pandemic. Our revised research question now explicitly asks: “What are the types, characteristics, and implications of intra-household gender disparities identified during the COVID-19 pandemic in LMICs in the MENA region?” (Lines 128-132).
